# Robust Shelf Monitoring Using Supervised Learning for Improving On-Shelf Availability in Retail Stores †

**DOI:** 10.3390/s19122722

**Published:** 2019-06-17

**Authors:** Kyota Higa, Kota Iwamoto

**Affiliations:** Data Science Research Laboratories, NEC Corporation, Kawasaki, Kanagawa 211-8666, Japan; k-iwamoto@ay.jp.nec.com

**Keywords:** image processing, change detection, change classification, background subtraction, convolutional neural network, on-shelf availability, product amount, surveillance camera, retail

## Abstract

This paper proposes a method to robustly monitor shelves in retail stores using supervised learning for improving on-shelf availability. To ensure high on-shelf availability, which is a key factor for improving profits in retail stores, we focus on understanding changes in products regarding increases/decreases in product amounts on the shelves. Our method first detects changed regions of products in an image by using background subtraction followed by moving object removal. It then classifies the detected change regions into several classes representing the actual changes on the shelves, such as “product taken (decrease)” and “product replenished/returned (increase)”, by supervised learning using convolutional neural networks. It finally updates the shelf condition representing the presence/absence of products using classification results and computes the product amount visible in the image as on-shelf availability using the updated shelf condition. Three experiments were conducted using two videos captured from a surveillance camera on the ceiling in a real store. Results of the first and second experiments show the effectiveness of the product change classification in our method. Results of the third experiment show that our method achieves a success rate of 89.6% for on-shelf availability when an error margin is within one product. With high accuracy, store clerks can maintain high on-shelf availability, enabling retail stores to increase profits.

## 1. Introduction

To improve profits, retail stores, such as supermarkets and convenience stores, should aim to reduce lost sales opportunities. One of the criteria for measuring loss of sales opportunities is on-shelf availability, which is generally defined as the availability of products for sale to shoppers, in the place they expect them and at the time they want to buy them [1,2]. When shoppers see a desired product to be out of stock (i.e., it has no on-shelf availability), the shoppers purchase the same or similar product at a competing retailer in the worst case. Therefore, on-shelf availability is a key factor for improving profits in retail stores. For further improving profits, products need to be stocked in places visible to the shoppers, that is, not at the back but on the front of shelves. In this paper, we define high on-shelf availability as a condition where a sufficient amount of products is stocked on the front of the shelves visible to the shoppers.

For maintaining high on-shelf availability, store clerks must often look around a store and replenish products or rearrange the shelves. However, this is labor-intensive. For effectively maintaining high on-shelf availability, a method is needed to automatically detect the amount of products on the front of the shelves, which frequently changes and to alert the store clerks to replenish products or rearrange the shelves when the product amount on the shelves is low.

We need to know the number of products on the shelves for accurately understanding the product amount. A check-out system shows the number of remaining products in the inventory. However, even if products are in the inventory, they might not reach the shelves due to inefficient shelf management. In other words, they are not on the shelves but in the store backroom. Hence, we cannot accurately understand the product amount on the shelves by using current check-out systems.

On the other hand, radio frequency identification (RFID) tags help us to understand the product amount on the shelves since they accurately detect the products in real-time. We can maintain high on-shelf availability since we can replenish products before they go out of stock. However, the RFID-based approach faces the problems of the exponential cost of sensor installation and time-consuming work of attaching tags to each product and removing them again at a billing counter (i.e., it is a high-cost solution).

**Figure 1:**
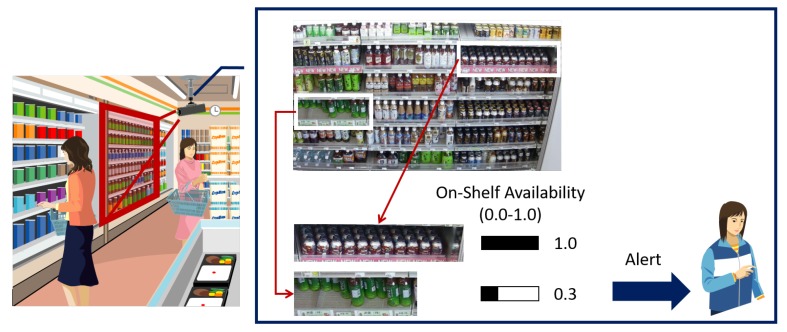
Overview of our target application to monitor the shelves in retail stores using a surveillance camera for maintaining high on-shelf availability.

For a low-cost solution, we apply an image processing technology to an image of the shelves. Figure 1 shows an overview of our target application to monitor the shelves in retail stores using a video captured from a surveillance camera on the ceiling for maintaining high on-shelf availability. It estimates the product amount on the shelves visible in the image and alerts the store clerks to replenish products or rearrange the shelves when the product amount on the shelves is low.

Methods [3,4] have recently been proposed to monitor the shelves by using the images for retail stores. Kejriwal et al. [3] proposed a method to directly obtain product count from images using mobile robots for stock monitoring and assessment in retail stores. This method identifies products on the shelves by feature matching with SURF (Speeded-Up Robust Features) [5] using product templates. Hence, it requires a large database of high-quality product templates, which is labor-intensive to maintain since products frequently change due to new and seasonal products arriving. Rosado et al. [4] proposed a method to detect out-of-stock regions in grocery retail shelves without using a large database. This method detects out-of-stock regions using points of interest (keypoints) detected by FAST (Features from Accelerated Segment Test) [6]. However, it cannot detect keypoints on products located on the front of the shelves when shoppers stand in front of the shelves, which leads to inaccurate estimation of the product amount on the shelves. Therefore, it cannot robustly monitor the shelves for maintaining high on-shelf availability.

This paper proposes a method to monitor the shelves robust to shopper occlusion and without a database of product templates for high on-shelf availability (this paper is an extended version of a previous paper [7]).

## 2. Related Work

Several methods have been proposed to identify products on shelves from an image or to enhance the accuracy of the reconstruction of large-scale objects from images. Liu et al. [8,9] proposed a method to check planogram (product layout) compliance by using recurring patterns. Product layout information is extracted from one single input image by unsupervised recurring pattern detection and matched via graph matching with the expected product layout. Saran et al. [10] proposed a visual analysis based framework using SURF for automated planogram compliance check in retail stores. These methods [8,9,10] focus on recognizing a planogram from an image. Cali and Ambu [11] proposed a method to improve the quality of the 3D digital surface reconstruction of large-scale objects from images. It focuses on reconstructing the surface of objects.

Kejriwal et al. [3] proposed a method to directly obtain product count from images captured using a monocular camera mounted on a mobile robot for stock monitoring and assessment in retail stores. Moorthy et al. [12] proposed a method to detect and count the front-facing products on the shelves as well as identify out-of-stock regions. These methods [3,12] identify products on the shelves by extracting SURF features around keypoints and by comparing them with those of product templates. Satapathy et al. [13] proposed a method to detect products on the shelves by using exhaustive template matching for raising alerts to replenish products. Bao et al. [14] proposed a method to accurately identify and locate a large number of objects in an image by using a scalable and compact binary local descriptor, named the BRIGHT (Binary ResIzable Gradient HisTogram) descriptor [15]. Zhang et al. [16] proposed a dual-layer density estimation-based architecture to detect multiple object instances from an image for robot inventory management. It identifies multiple object instances by feature matching with SIFT (Scale-Invariant Feature Transform) [17]. Scovanner et al. [18] proposed a 3-dimensional SIFT descriptor for video or 3D imagery. Scale and rotation invariant local descriptors, such as SIFT, SURF and BRIGHT, have enabled identification of real-world objects in images with robustness against differing camera viewpoints, occlusions and lighting conditions. To achieve high performance, these methods [3,12,13,14,16,18] require a large database of high-quality product templates that must be updated every time products on the shelves change. However, the large database is time-consuming to maintain since the products on the shelves frequently change due to new and seasonal products arriving.

On the other hand, a method has been proposed to detect out-of-stock regions without a large database of high-quality product templates. Rosado et al. [4] proposed a supervised learning approach for out-of-stock detection from high-resolution panoramic images of grocery retail shelves. This method detects out-of-stock regions on the basis of the density of keypoints detected by FAST since those regions are more homogeneous in terms of color and texture than product regions, that is, a sufficient number of keypoints cannot be detected from out-of-stock regions. Detecting keypoints from the images needs no database of product templates. However, we cannot accurately understand the product amount on the front of the shelves by using this method since it detects keypoints on products located in the second row of the shelves even when shoppers take products from the front of the shelves. In addition, when shoppers stand in front of the shelves, it cannot detect keypoints on products located on the front of the shelves, which also leads to inaccurate estimation of the product amount on the shelves. Therefore, it cannot robustly monitor the shelves for maintaining high on-shelf availability.

Thus, there is still no method to easily and robustly monitor the shelves from the image for high on-shelf availability.

## 3. Proposed Method

### 3.1. Overview

**Figure 2:**
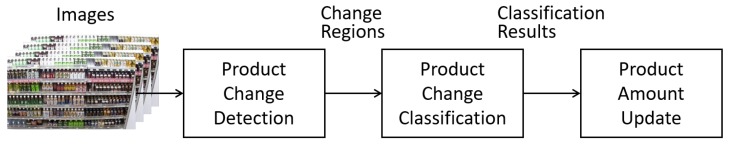
Process flow of proposed method.

The proposed method robustly monitors the shelves by detecting and classifying changes in products regarding increases/decreases in product amount, such as “product taken (decrease)” and “product replenished/returned (increase)”, using images from a surveillance camera and by updating the shelf condition representing presence/absence of products on the front of the shelves from the initial shelf condition given in advance. Background subtraction (or foreground detection) [19,20,21], which is a widely used approach for detecting changes in images as foregrounds, cannot distinguish between “product taken” and “product replenished/returned” since it detects regions that do not match a reference image, often called “background image” or “background model”. Hence, background subtraction alone is not sufficient for robustly monitoring the shelves for maintaining high on-shelf availability. To solve this problem, we need to classify the changes in the image into the actual changes in products on the shelves, such as “product taken” and “product replenished/returned”. The process flow of our method consists of three blocks as shown in Figure 2. It first detects change regions of the shelves in the image by background subtraction on the basis of statistical information of pixels. To reduce false positives of change regions such as shoppers, it tracks detected foregrounds between consecutive images and removes them on the basis of their moving distance. It then classifies the detected change regions into four classes representing the actual changes on the shelves by supervised learning using convolutional neural networks (CNNs) [22]. It finally updates the shelf condition representing presence/absence of products on the front of the shelves using classification results and computes the product amount visible in the image as on-shelf availability.

### 3.2. Detection of Product Changes

We accurately detect only the change regions of products on the shelves by background subtraction followed by moving object removal. Our method detects the change regions in images as foregrounds by using the background subtraction [19]. However, background subtraction detects not only the change regions of products but also false positives such as moving objects (i.e., shoppers and store clerks). Therefore, we track the foregrounds between consecutive images and remove them on the basis of their moving distance. The foregrounds with a moving distance larger than $T_{d}$ pixels are removed as moving objects, whereas the static foregrounds detected for more than $T_{s}$ seconds are selected as the change regions of products.

The Hungarian method [23], which is a combinatorial optimization algorithm that solves the assignment problem, is used for tracking the foregrounds in the consecutive images. In particular, the foregrounds are tracked by minimizing the summation of assignment cost among *N* foregrounds detected in a current image and *M* foregrounds detected in a previous image. The cost matrix of the Hungarian method used in our method is as follows:

(1) $$C_{i,j}=\left[\begin{array}{ccc} c_{1,1} & \cdots & c_{1,N}\\ \vdots & \ddots & \vdots \\ c_{M,1} & \cdots & c_{M,N}\\ c_{def} & & c_{max}\\ & \ddots & \\ c_{max} & & c_{def} \end{array}\right].$$

Here, the parameters $c_{def}$ and $c_{max}$ are a default value and a value sufficiently larger than the default value, respectively.

The assignment cost $c_{i,j}$ consists of the similarity of the color histograms in the foregrounds, area ratio of the foregrounds and aspect ratio of bounding rectangles of the foregrounds.

The similarity cost $c_{i,j}^{color}$ is computed as,

(2) $$c_{i,j}^{color}=1.0-\frac{\Sigma_{k}\left(H_{t}^{j}\left(k\right)-{\bar{H}}_{t}^{j}\right)\left(H_{t-1}^{i}\left(k\right)-{\bar{H}}_{t-1}^{i}\right)}{\sqrt{\Sigma_{k}{\left(H_{t}^{j}\left(k\right)-{\bar{H}}_{t}^{j}\right)}^{2}\Sigma_{k}{\left(H_{t-1}^{i}\left(k\right)-{\bar{H}}_{t-1}^{i}\right)}^{2}}},$$

(3) $${\bar{H}}_{m}^{n}=\frac{1}{K}\Sigma_{k}H_{m}^{n}\left(k\right),$$

where $H_{t}^{j}$ and $H_{t-1}^{i}$ are color histograms of the *j*th and *i*th foregrounds in the current and previous images, respectively. *K* is the number of bins. Note that the total area of a histogram has been normalized to 1.0.

The area ratio cost $c_{i,j}^{area}$ is computed as,

(4) $$c_{i,j}^{area}=1.0-\left\{\begin{array}{cc} S_{t}^{j}/S_{t-1}^{i} & \mathrm{if}\;S_{t}^{j}<S_{t-1}^{i}\\ S_{t-1}^{i}/S_{t}^{j} & \mathrm{else} \end{array}\right.,$$

where $S_{t}^{j}$ and $S_{t-1}^{i}$ are areas of the *j*th and *i*th foregrounds in the current and previous images, respectively.

The aspect ratio cost $c_{i,j}^{aspect}$ is computed as,

(5) $$c_{i,j}^{aspect}=1.0-\left\{\begin{array}{cc} A_{t}^{j}/A_{t-1}^{i} & \mathrm{if}\;A_{t}^{j}<A_{t-1}^{i}\\ A_{t-1}^{i}/A_{t}^{j} & \mathrm{else} \end{array}\right.,$$

where $A_{t}^{j}$ and $A_{t-1}^{i}$ are aspect ratio of the bounding rectangle for the *j*th and *i*th foregrounds in the current and previous images, respectively.

Finally, the assignment cost $c_{i,j}$ is computed as,

(6) $$c_{i,j}=w_{1}\times c_{i,j}^{color}+w_{2}\times c_{i,j}^{area}+w_{3}\times c_{i,j}^{aspect},$$

where *w* means weight.

**Figure 3:**
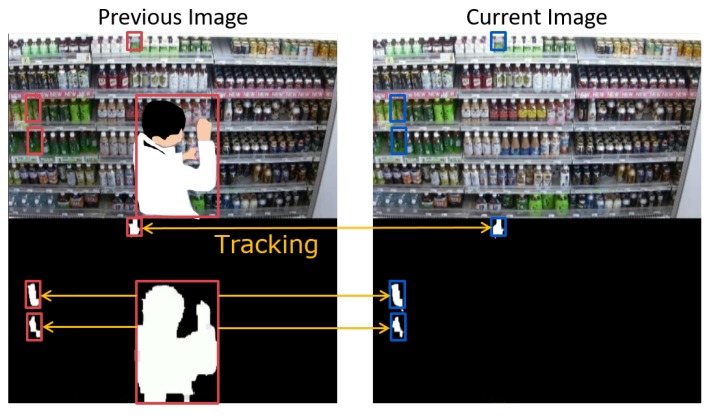
Example of detecting and tracking foregrounds.

Figure 3 shows an example of detecting and tracking the foregrounds. Left and right images show the previous and current images, respectively. Top and bottom images show an input image and the foregrounds, respectively. Note that the person in the top-left image is retouched for privacy protection. Here, three small foregrounds and one large foreground are detected in the previous image and three small foregrounds are detected in the current image. Our method accurately detects only the changes in products even when the shoppers stand in front of the shelves by tracking the foregrounds between the consecutive images on the basis of three similarities by using the Hungarian method.

### 3.3. Classification of Product Changes

The detected change regions are classified into four classes representing the actual changes on the shelves by supervised learning using CNNs for accurately understanding increases/decreases in the product amount.

**Figure 4:**
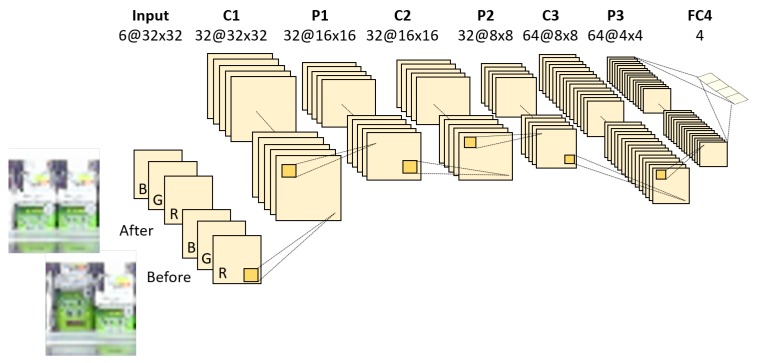
Network architecture based on CIFAR-10 (Canadian Institute For Advanced Research). It consists of three convolutional layers, three pooling layers and one fully-connected layer.

**Figure 5:**
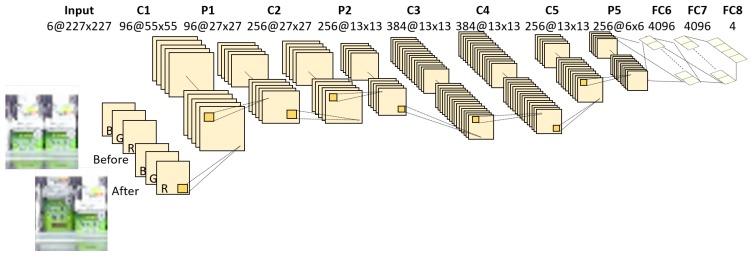
Network architecture based on CaffeNet. It consists of five convolutional layers, three pooling layers and three fully-connected layers.

**Figure 6:**
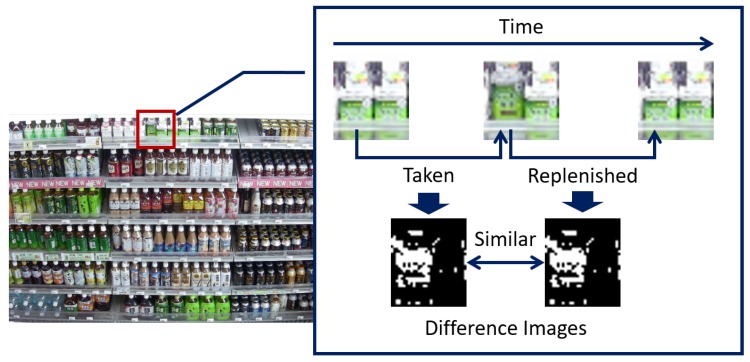
Examples of difference images for change regions of “product taken” and “product replenished”.

In this paper, we try two networks for classifying the detected change regions: a CIFAR-10-based network shown in Figure 4 and a CaffeNet-based network (http://caffe.berkeleyvision.org/) shown in Figure 5. The input and final fully-connected layers in both networks are modified compared with those of the original networks. These networks are learned using training samples collected from videos taken in real stores. Input data of our network are six-channel images constructed from images before and after the change as shown in Figure 4 and Figure 5. Zhao et al. [24] proposed a method to classify difference images by deep neural networks. However, it cannot distinguish the actual changes on the shelves such as “product taken” and “product replenished/returned” since they are essentially symmetrical operations (i.e., the difference images of those change regions are almost identical) as shown in Figure 6. Therefore, we use the six-channel images to which no extra operations are applied as input data of our network. Note that the images before and after the change are resized to 32×32 pixels for the CIFAR-10-based network or 227×227 pixels for the CaffeNet-based network before constructing the six-channel image.

**Figure 7:**
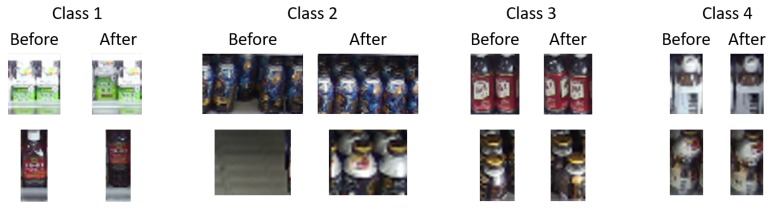
Examples of images for each class in product change classification.

Our network classifies the detected change regions into four classes shown in Table 1. Examples of images for each class are shown in Figure 7. Classes 1 and 2 show “product taken” and “product replenished/returned”, respectively. Class 3 shows that the position or direction of a product slightly changed since a shopper touched it. Class 4 shows false positives unrelated to human movement, such as illumination changes. Classes 1 and 2 show that the product amount on the shelves decreases or increases, respectively, whereas classes 3 and 4 show that the product amount on the shelves does not change.

### 3.4. Update of Product Amount

Our method updates the shelf condition representing presence/absence of products on the front of the shelves using the classification results and computes the product amount visible in the image as on-shelf availability using the updated shelf condition and predefined monitoring areas.

**Figure 8:**
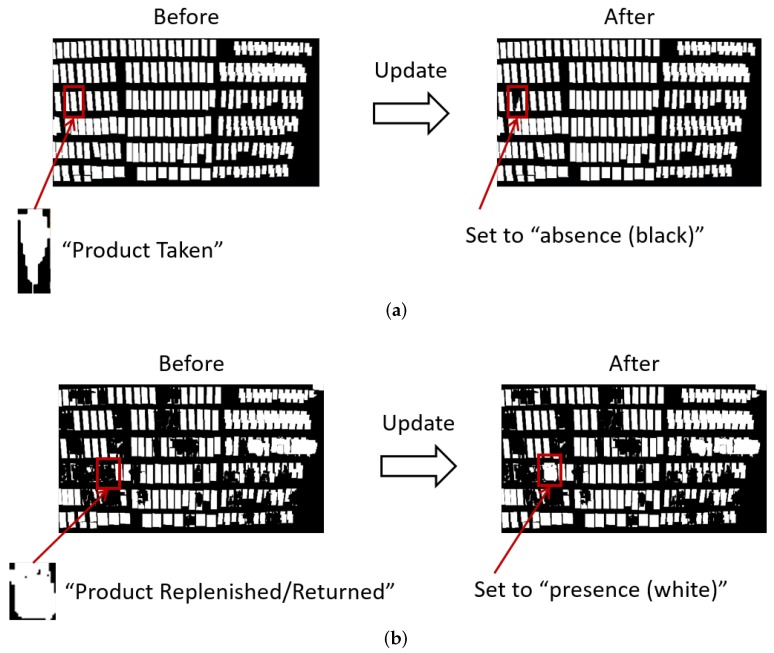
Examples of shelf condition represented as binary image. (**a**) “Product taken”. (**b**) “Product replenished/returned”.

Figure 8 shows examples of the shelf condition represented as a binary image. Left and right images are the shelf condition before and after an update, respectively. The white and black regions of the shelf condition show “presence” and “absence” of products, respectively. When the classification results are “product taken (class 1)”, the shelf condition corresponding to the change regions is set to “absence (black)” as shown in Figure 8a. When the classification results are “product replenished/returned (class 2)”, the shelf condition corresponding to the change regions is set to “presence (white)” as shown in Figure 8b. The shelf condition is not changed in the case of other classification results (classes 3 and 4).

**Figure 9:**
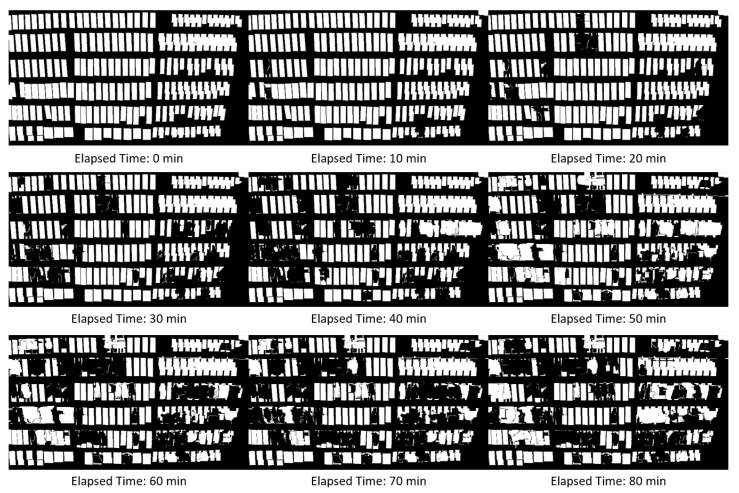
Examples of shelf condition updated by our method.

Figure 9 shows examples of the shelf condition updated by our method. The shelf condition at 0 min shows the initial shelf condition given in advance. As shown in Figure 9, our method updates the shelf condition, which frequently changes on the basis of “product taken” and “product replenished/returned” detected from the image.

**Figure 10:**
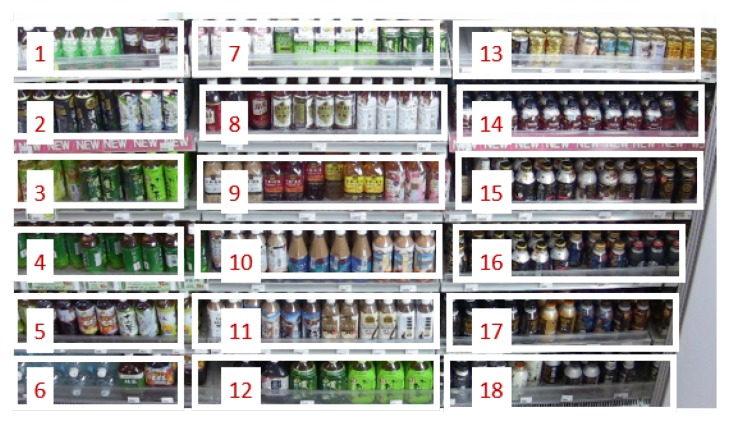
Predefined monitoring areas with shelf number.

**Figure 11:**
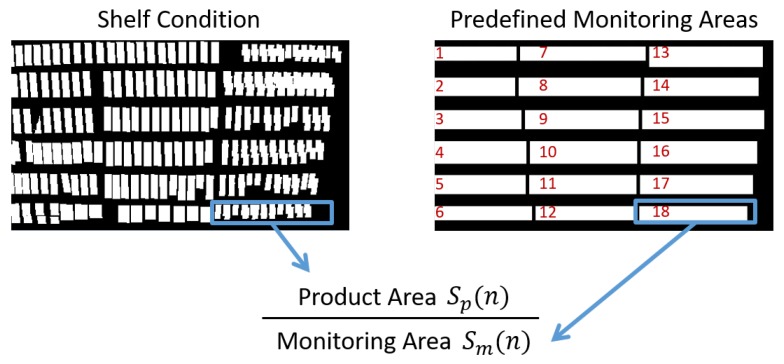
Overview of computing on-shelf availability for *n*th shelf.

After updating the shelf condition, our method computes on-shelf availability (product amount) visible in the image between the range of 0.0 and 1.0 using the predefined monitoring areas shown in Figure 10 as follows,

(7) $$OSA\left(n\right)=S_{p}\left(n\right)/S_{m}\left(n\right),$$

where OSA(n) is on-shelf availability for the *n*th shelf. $S_{p}\left(n\right)$ and $S_{m}\left(n\right)$ are the product areas of the *n*th shelf (i.e., the number of white pixels in the shelf condition corresponding to the *n*th shelf) and *n*th monitoring area (i.e., the number of pixels in the *n*th monitoring area), respectively. Figure 11 shows an overview of computing on-shelf availability for the *n*th shelf. When some products are taken/replenished simultaneously as shown in the lower example of class 2 in Figure 7, on-shelf availability increases/decreases on the basis of the summation of changed areas.

## 4. Experiments

### 4.1. Experimental Conditions

We conducted three experiments on two videos captured from a fixed camera on the ceiling in a real store for evaluating the accuracy of our method. Table 2 and Table 3 show details of the videos used in the experiments and parameters in the product change detection, respectively. The videos of shelves with bottled beverages shown in Figure 10 were used in the experiments and showed more than 200 shoppers in total. As shown in Table 2, the resolution and frame rate of the videos are low, which makes it difficult to track moving objects between consecutive images. However, such conditions are suitable for retailers since they decrease costs of data storage to save videos. The networks for the product change classification shown in Figure 4 and Figure 5 were learned using training samples extracted from other videos taken in the real store with the same camera conditions. The number of training samples for each class is shown in Table 4. The initial shelf condition shown in Figure 8 and the monitoring areas shown in Figure 10 were set manually. For every minute, we computed on-shelf availability (i.e., product amount on shelves) using Equation (7) and then counted the number of successful frames whose difference between the estimated on-shelf availability OSA(n) and ground truth is less than an error margin. The success rate for each shelf is computed as,

(8) $$SuccessRate=S_{n}/\left(S_{n}+U_{n}\right),$$

where $S_{n}$ and $U_{n}$ are the numbers of successful and unsuccessful frames for the *n*th shelf, respectively.

### 4.2. Experiment 1: Comparison of Networks for Product Change Classification

**Figure 12:**
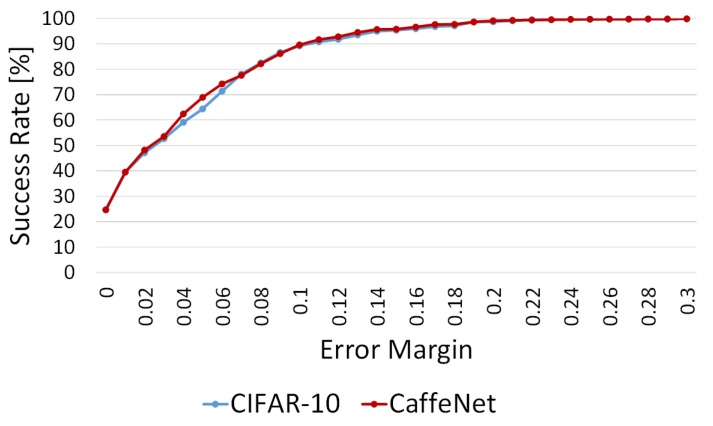
Average success rate for on-shelf availability for all the shelves at various error margins in experiment 1.

We evaluated the estimation accuracy of the CIFAR-10-based network and the CaffeNet-based network for selecting the network for the product change classification. Figure 12 shows the average success rate for on-shelf availability for all the shelves at various error margins. The vertical and horizontal axes are the success rate and the error margin, respectively. The blue and red lines show the success rates of the CIFAR-10-based and CaffeNet-based networks, respectively. As shown in Figure 12, the two networks barely differ in terms of the success rates for on-shelf availability, which means that the network for the product change classification can be selected on the basis of hardware resources such as memory size or system requirements such as processing time. We select the CaffeNet-based network for the product change classification since its success rate is slightly higher than that of the CIFAR-10-based network although it needs much memory (i.e., the model size is large).

### 4.3. Experiment 2: Evaluation of Effectiveness for Product Change Classification

**Figure 13:**
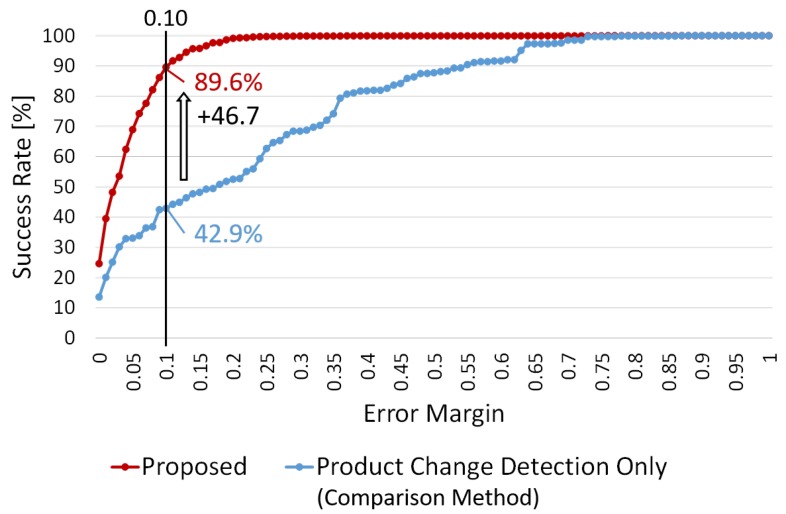
Average success rate for on-shelf availability for all the shelves at various error margins in experiment 2.

For evaluating the effectiveness of the product change classification in our method, we compared the estimation accuracy of our method with that of a method accumulating the product change regions detected by background subtraction [19] (i.e., the product change detection only). The comparison method accumulates all the detected change regions on the shelves as “product taken”. Figure 13 shows the average success rate for on-shelf availability for all the shelves at various error margins in this experiment. The red and blue lines show the success rates of the proposed and comparison methods, respectively. Our method achieves the success rate of 89.6% for on-shelf availability at the error margin of 0.10, which is equivalent to an error of approximately ±1 product or less since approximately 10 products are on each shelf as shown in Figure 10. Our method improves the success rate by 46.7 points compared with that of the comparison method (42.9%) at the error margin of 0.10, which shows the effectiveness of the product change classification by supervised learning using CNNs in our method.

### 4.4. Experiment 3: Comparison of Proposed and Conventional Methods

**Figure 14:**
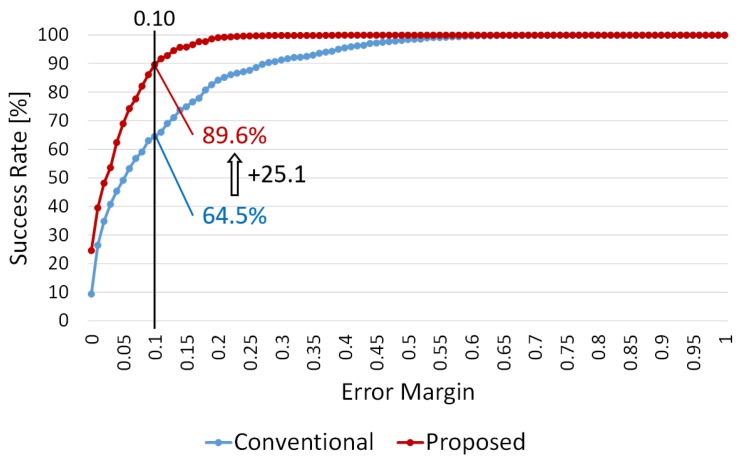
Average success rate for on-shelf availability for all the shelves at various error margins in experiment 3.

We compared the estimation accuracy of the proposed method with that of the conventional method [4], which is the state-of-the-art technology of out-of-stock detection. Figure 14 shows the average success rate for on-shelf availability for all the shelves at various error margins in this experiment. The red and blue lines show the success rates of the proposed and conventional methods, respectively. Our method (89.6%) improves the success rate by 25.1 points compared with that of the conventional method (64.5%) at the error margin of 0.10.

Table 5 shows the success rate for each shelf in our method at the error margin of 0.10. High (over 90%) and low (under 80%) success rates are written in red in bold and blue with an underline, respectively. As shown in Table 5, our method achieves high success rates for 10 out of 18 shelves at the error margin of 0.10.

**Figure 15:**
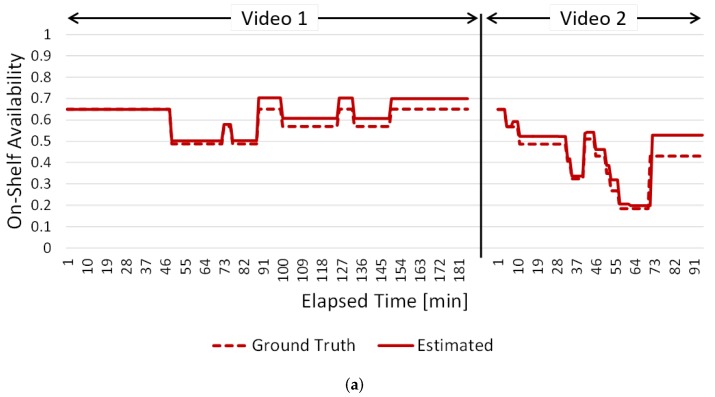

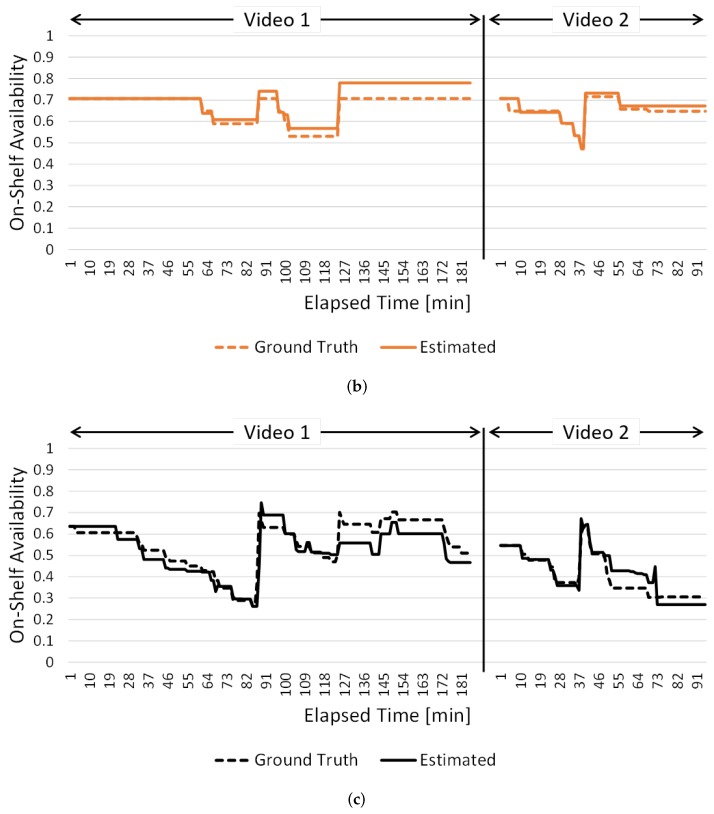
Changes in on-shelf availability every minute in cases of high success rates. (**a**) Results for shelf #3. (**b**) Results for shelf #7. (**c**) Results for shelf #17.

**Figure 16:**
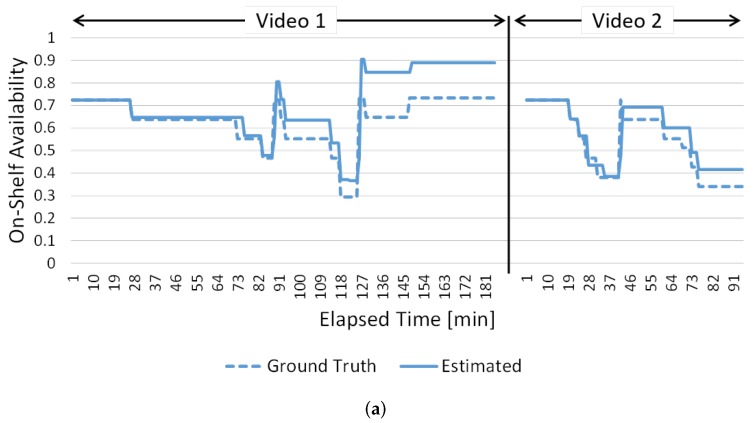

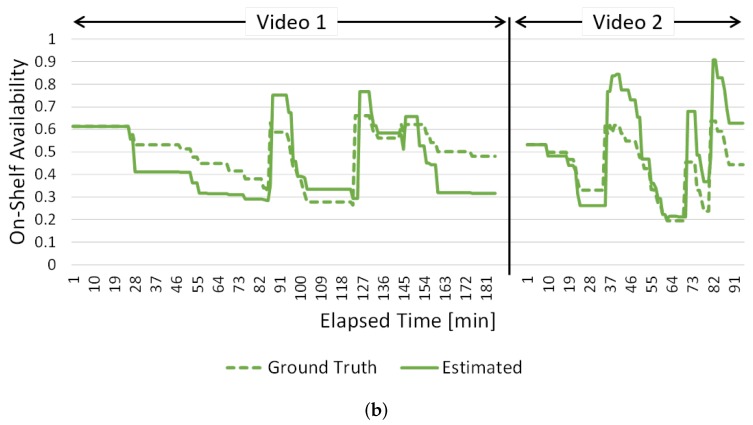
Changes in on-shelf availability every minute in cases of low success rates. (**a**) Results for shelf #1. (**b**) Results for shelf #15.

Figure 15 and Figure 16 show the changes in on-shelf availability every minute. The results for shelves #3, #7 and #17 are shown in Figure 15 as examples of high success rates. The results for shelves #1 and #15 are shown in Figure 16 as examples of low success rates. The vertical and horizontal axes are on-shelf availability and elapsed time of the videos, respectively. Dotted and solid lines show ground truth and the estimated on-shelf availability, respectively.

### 4.5. Discussion

Figure 15 shows that our method accurately tracks the changes in on-shelf availability since the increases/decreases in on-shelf availability estimated by our method (solid lines) are very similar to those of the ground truth (dotted lines).

**Figure 17:**
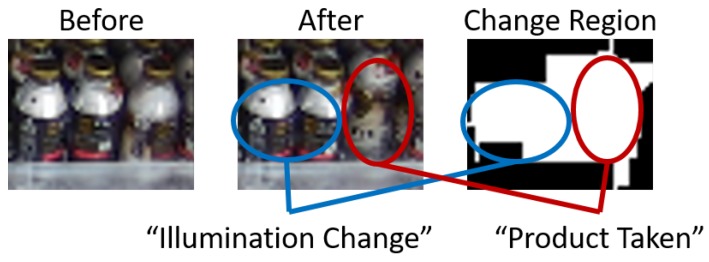
Example in which different kinds of change regions are detected as one change region since those regions adjoin.

Figure 16 shows some points to be improved. In the results for shelf #1 shown in Figure 16a, our method fails to estimate on-shelf availability at 127 min in video 1 due to a classification error; specifically, on-shelf availability estimated by our method increases more than the ground truth. Since our method tracks relative changes in on-shelf availability, the classification error affects estimation of on-shelf availability after 127 min. The estimation accuracy can be improved by introducing a scheme to regularly reset the accumulated error. In the results for shelf #15 shown in Figure 16b, our method fails to estimate on-shelf availability several times. For example, at 27 and 161 min in video 1, two kinds of change regions (“product taken” and “illumination change”) were detected as one change region as shown in Figure 17 since those regions adjoin and then the detected change region was classified into “product taken”. Hence, our method estimated that on-shelf availability had decreased significantly at 27 and 161 min in video 1. This problem can be expected to be solved by dividing the detected change region in detail by using an image segmentation method and by classifying the divided regions.

**Figure 18:**
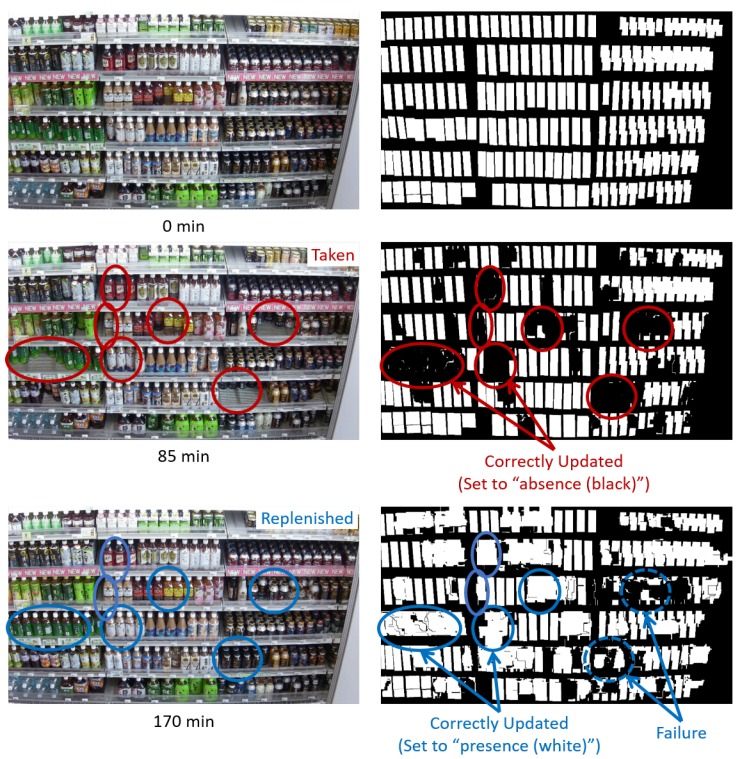
Some results for updated shelf condition in video 1.

**Figure 19:**
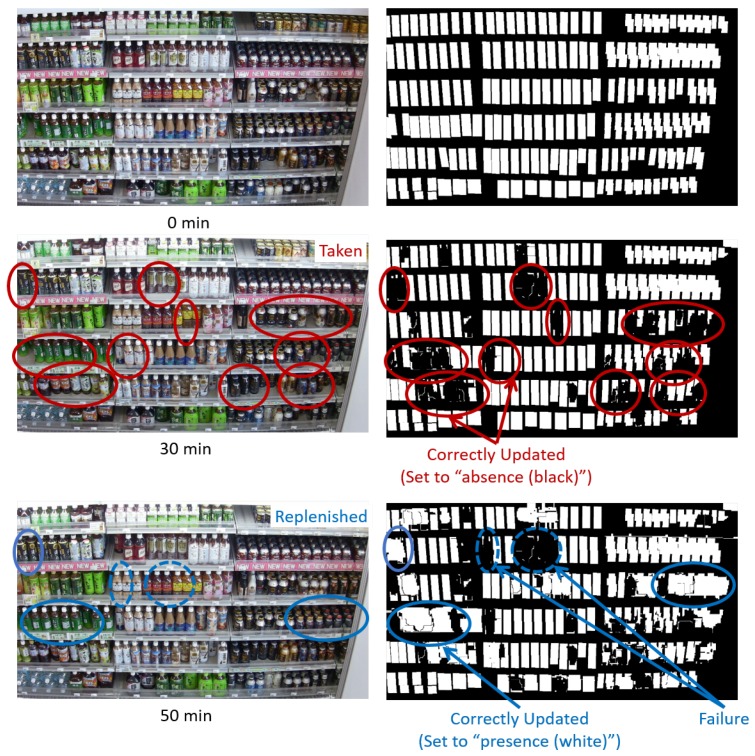
Some results for updated shelf condition in video 2.

Figure 18 and Figure 19 show some results for the updated shelf condition in videos 1 and 2. The first and second rows show the captured images and the display condition (i.e., presence/absence of products), respectively. Some regions in the images are marked as examples of “product taken” and “product replenished”. This shows that our method can track the changes in products on the shelves regarding increases/decreases in the product amount. These results suggest that our method monitors the shelves from the images robust to shopper occlusion and without the database of product templates for maintaining high on-shelf availability.

**Figure 20:**
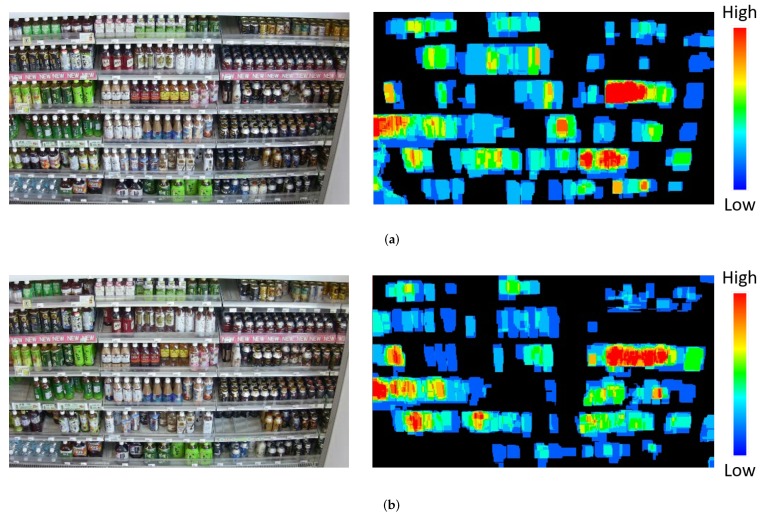
Examples of another application using our method. (**a**) Result for video 1. (**b**) Result for video 2.

Figure 20 shows examples of another application using our method. Left and right images are a captured image and a heatmap, respectively. Each heatmap shows the accumulation of the product changes detected from the images. In other words, it shows which shelves shoppers frequently accessed. By visualizing such information, retailers can plan a suitable planogram (product layout) for increasing their profits (e.g., a planogram to reduce low-accessed shelves and a planogram to increase the number of products displayed on high-accessed shelves).

## 5. Conclusions

This paper proposed a method to robustly monitor shelves in retail stores using supervised learning for improving on-shelf availability. To ensure high on-shelf availability, which is a key factor for improving profits in retail stores, we focused on understanding changes in products regarding increases/decreases in product amount on the shelves. Our method first accurately detects only change regions of products in an image by using background subtraction followed by moving object removal for removing detected change regions unrelated to the changes of products (i.e., shoppers and store clerks). To reduce the moving objects, we track detected foregrounds between consecutive images and remove them on the basis of their moving distance. The detected change regions are then classified into four classes representing the actual changes on the shelves, such as “product taken (decrease)” and “product replenished/returned (increase)”, by supervised learning using convolutional neural networks for accurately understanding increases/decreases in the product amount. Finally, the shelf condition representing presence/absence of products on the front of the shelves is accurately updated using classification results and the product amount visible in the image is computed as on-shelf availability using the updated shelf condition and predefined monitoring areas. Three experiments were conducted using two videos captured from a surveillance camera on the ceiling in a real store. The results of the first and second experiments show the effectiveness of the product change classification using a CaffeNet-based network in our method. The results of the third experiment show that the proposed method achieves a success rate of 89.6% for on-shelf availability when an error margin is within one product, which is 25.1 points higher than that of a conventional method (64.5%). With high accuracy, store clerks can maintain high on-shelf availability, enabling retail stores to increase their profits.

## Figures and Tables

**Table 1:** Output of our network in product change classification.

Class	Description	Product Amount
1	A product was taken.	decrease
2	A product was replenished by store clerks or returned by shoppers.	increase
3	A position or direction of a product slightly changed since a shopper touched it.	no change
4	It was a false positive unrelated to human movement, such as an illumination change.	no change

**Table 2:** Details of videos used in experiments.

Video Length	Video 1	185 min
	Video 2	95 min
**Resolution**		480 × 270 pixels (1/16 of Full HD)
**Frame Rate**		1 fps

**Table 3:** Details of parameters in product change detection.

$T_{d}$ ***in Section 3.2***	10 pixels
$T_{s}$ ***in Section 3.2***	30 s
$c_{def}$ ***in Equation (1)***	1.0
*K* ***in Equation (3)***	32
*w* ***in Equation (6)***	1.0

**Table 4:** Number of training samples in product change classification.

Class	Training Samples	Class	Training Samples
***1***	16,038	***3***	1448
***2***	16,038	***4***	3486

**Table 5:** Success rate for each shelf at error margin of 0.10.

Shelf Number	Success Rate	Shelf Number	Success Rate	Shelf Number	Success Rate
***1***	77.7%	***7***	**100%**	***13***	**99.3%**
***2***	80.6%	***8***	83.3%	***14***	**100%**
***3***	**99.6%**	***9***	**97.1%**	***15***	51.4%
***4***	87.4%	***10***	**98.2%**	***16***	**90.3%**
***5***	80.6%	***11***	88.8%	***17***	**96.0%**
***6***	**99.6%**	***12***	**99.3%**	***18***	83.5%
